# Оптимизация комплексного подхода к коррекции голоса при эндокринопатиях (аналитический обзор)

**DOI:** 10.14341/probl12822

**Published:** 2022-04-30

**Authors:** С. В. Старостина, Я. А. Стаценко, В. М. Свистушкин

**Affiliations:** Первый Московский государственный медицинский университет им. И.М. Сеченова (Сеченовский университет); Первый Московский государственный медицинский университет им. И.М. Сеченова (Сеченовский университет); Первый Московский государственный медицинский университет им. И.М. Сеченова (Сеченовский университет)

**Keywords:** гортань, эндокринопатия, голос

## Abstract

Дисфония — симптом множества эндокринных патологий. Охриплость, огрубение голоса, снижение его высоты, сужение диапазона, утомляемость — характерные жалобы при гипотиреозе, обусловленные увеличением количества полисахаридов и накоплением жидкости в собственной пластинке голосовых складок. Избыток соматотропного гормона вызывает гиперпродукцию инсулиноподобного фактора роста-1, что приводит к аномалиям черепно-лицевой области и пролиферации тканей верхних дыхательных путей, в том числе утолщению хрящей гортани, голосовых складок и снижению высоты голоса. Гипергликемия, изменение баланса жидкости и электролитов при сахарном диабете могут косвенно влиять на голос: ксеростомия затрудняет фонацию в связи с нарушением увлажнения слизистой оболочки гортани, а диабетическая нейропатия нередко нарушает работу мышц гортани, участвующих в голосообразовании. Изменение голоса наблюдается не только при эндокринных нарушениях, но и во время полового созревания, фаз менструального цикла, в период менопаузы. Ларингеальные структуры модифицируются под влиянием гормонов и  подвергаются внешним воздействиям, что в целом изменяет высоту и интенсивность голоса, тембр и резонанс, артикуляционные и просодические характеристики. Представленный обзор направлен на обобщение и систематизацию данных литературы, касающихся изменений голоса при ряде физиологических и патологических состояний у пациентов различных возрастных групп и пола. Показаны возможности мультидисциплинарного подхода к рациональной коррекции голоса.

## Abstract (en)

Dysphonia is a symptom of many endocrine pathologies. Hoarseness, voice fatigue, decrease of the voice pitch, reduce of the range are typical complaints for hypothyroidism, caused by an increased amount of polysaccharides and accumulation of fluid in the lamina propria of the vocal folds. An excess of growth hormone causes an overproduction of insulin-like growth factor-1, which leads to abnormalities in the craniofacial region and proliferation of the upper respiratory tract tissues, including thickening of the laryngeal cartilages, vocal folds and decreasing of the voice pitch. Hyperglycemia, changes in the balance of fluid and electrolytes in diabetes mellitus can indirectly affect the voice: xerostomia complicates phonation due to impaired hydration of the laryngeal mucosa, diabetic neuropathy often disrupts the work of the laryngeal muscles involved in voice formation. Voice changes are observed not only in endocrine disorders, but also during puberty, the phases of the menstrual cycle and during menopause. Laryngeal structures are modified under the influence of hormones and external factors, which generally changes pitch and intensity of the voice, timbre and resonance, articulation and prosodic characteristics. The purpose of this review is to summarize and systematize data on physiological and pathological voice changes in patients of different age groups and sex. The possibilities of a multidisciplinary approach to rational voice correction are demonstrated.

## ВВЕДЕНИЕ

Голос является вторичным, но очень важным половым признаком. На протяжении всей жизни голосовой аппарат человека модифицируется под воздействием гормонов. Изменение голоса наблюдается не только при эндокринных нарушениях, в том числе социально значимых, таких как новообразования щитовидной железы, сахарный диабет, но и во время полового созревания, фаз менструального цикла, в период менопаузы и при ряде других физиологических и патологических состояний. Несмотря на то что дисфония редко является первой и основной жалобой при эндокринных нарушениях, а инволюционные изменения голоса воспринимаются населением как возрастная норма, стоит уделить пристальное внимание данному вопросу, так как на современном этапе развития медицины целью лечения пациента является не только восстановление и поддержание здоровья, но также повышение качества жизни.

Цель данного исследования — оценить нарушения голоса при эндокринопатиях, возможности различных методов коррекции голоса по данным литературы, что поможет врачу и пациенту выбрать наиболее рациональный подход к решению проблемы.

Поиск литературы проводили в отечественных (CyberLeninka, Академия Google) и международных (PubMed) базах данных на русском и английском языках. Приоритетными были источники с 2016 по 2021 гг. Однако с учетом недостаточной изученности выбранной темы использовались и более ранние данные.

## Эндокринопатии и голос

Влияние эндокринной системы на голосовой аппарат было отмечено уже в эпоху средневековья, когда в Центральной Азии и в европейских странах была распространена практика кастрации юных певцов с исключительно хорошими голосами. Операция оберегала голосовой аппарат от изменений в ходе полового созревания, что продлевало профессиональную жизнь певца [1].

На сегодняшний день связь изменений голоса и гормонального влияния на голосовой аппарат очевидна. Ряд исследований доказывает наличие рецепторов к половым и тиреоидным гормонам на голосовых складках и прочих структурах гортани [2][3]. J. Abitbol и соавт. (1989) отметили, что мазки с шейки матки и гортани, окрашенные с целью выявления рецепторов к эстрогену во время фаз менструального цикла, микроскопически неразличимы [4]. Ларингеальные структуры модифицируются под влиянием и других гормонов, а также подвергаются внешним воздействиям, что в целом изменяет ряд характеристик голоса. Среди них — высота голоса, сила, тембр, резонанс, артикуляционные и просодические характеристики. Для акустических параметров голоса характерны гендерные различия. Определяющая величина — основная частота (F0), соответствующая числу колебаний голосовых складок в секунду (Гц), воспринимается как высота голоса. Голосовые складки у женщин короткие, тонкие, отличаются выраженным натяжением, что приводит к быстрому их колебанию и более высокому голосу по сравнению с мужчинами. Для женщин также характерен отличительный резонанс за счет короткого голосового тракта [1].

Для обозначения нарушений голоса различной этиологии используют два термина: «дисфония» и «афония». Дисфония — это расстройство голоса, а афония — полная утрата звучного голоса при сохранении шепота. Нормальный же голос должен быть приятным на слух, достаточно громким, обладать соответствующим рото-носовым резонансом, высота основного тона должна соответствовать возрасту и полу человека; темп речи должен быть таким, чтобы не нарушались выше приведенные основные субъективные характеристики голоса. Гортань является гормонозависимым органом, особенно чутко реагирующим на изменения уровня половых и тиреоидных гормонов [5].

Охриплость, огрубение голоса, снижение его высоты, сужение диапазона, утомляемость — характерные жалобы при гипотиреозе, обусловленные увеличением количества полисахаридов и накоплением жидкости в собственной пластинке голосовых складок, отеком перстнещитовидной мышцы и блуждающего нерва, парезом голосовых складок вследствие компрессии возвратного нерва увеличенной щитовидной железой. Данные симптомы прогрессируют медленно и нередко остаются незамеченными самим пациентом. Голос полностью восстанавливается в течение 3–6 мес после достижения эутиреоза и уменьшения объема щитовидной железы в результате рациональной гормональной терапии или после восстановления подвижности гортани.

В литературе встречаются клинические случаи с дисфонией и дизартрией в качестве ведущего симптома гипотиреоза. Пациентка Kawther T. El-Shafie с дизартрией в качестве основной жалобы, а также эпизодами дисфонии, дисфагии, храпа и апноэ во сне отметила быстрый регресс названных симптомов на фоне приема левотироксина [6] C. Stollberger и соавт. [7] описали пациента с гипотиреозом, предъявлявшим жалобы на нарушение голоса и речи. Вероятно, характерные для выраженного гипотиреоза снижение тонуса мышц голосового и речевого аппарата, макроглоссия и нарушение речевого дыхания проявились в виде нечеткости речи и гнусавости голоса. В анамнезе пациента дизартрии предшествовал синдром обструктивного апноэ во сне (СОАС), основной фактор риска развития которого — избыточная масса тела. Ожирение, как эндокринное заболевание, влияет на дыхание различными механизмами, включая местные изменения в структуре верхних дыхательных путей (измененная геометрия) или функции (увеличенный коллапс), а также за счет повышения внутрибрюшного давления (висцеральное ожирение). Литературные данные показывают, что у пациентов с СОАС без ожирения имеется избыточное депонирование жира в верхних дыхательных путях по сравнению с лицами без СОАС [8]. Жалобы на храп, сухость во рту по утрам из-за дыхания открытым ртом и изменение голоса (охриплость) весьма характерны для пациентов с выраженным ожирением и СОАС [9].

Дисфония может быть обусловлена непосредственным (механическим) воздействием на гортань увеличенной щитовидной железы (ЩЖ) либо повреждением структур гортани в случае инфильтративного процесса при злокачественном новообразовании (ЗНО) ЩЖ. К значительному увеличению объема железы могут приводить различные заболевания: узловой/многоузловой зоб, аутоиммунные процессы ЩЖ (тиреоидиты, болезнь Грейвса), кисты органа. Инфильтрирующая окружающие ткани карцинома ЩЖ, зоб либо единичные крупные образования, локализующиеся перед трахеей, могут проявляться голосовыми компрессионными симптомами, а также дисфагией и одышкой. При успешном хирургическом лечении без повреждения окружающих структур у 85% пациентов компрессионные симптомы исчезают [10].

Хирургия ЩЖ и околощитовидных желез (ОЩЖ) связана с риском постоянного или преходящего повреждения возвратного нерва, последующего пареза гортани и дисфонии. Последняя может наблюдаться и у пациентов без повреждения возвратного нерва, в связи с отеком или повреждением мышц гортани, постинтубационными травмами, ларинготрахеальной фиксацией и другими причинами [10]. Нарушение подвижности голосовых складок представляется симптомокомплексом стойких (паралич) или преходящих (парез) нарушений вследствие:

При односторонних парезах гортани и находящейся в интермедианной или парамедианной, реже в латеральной позиции, голосовой складке наблюдаются стойкие нарушения фонаторной функции — охриплость, битональность или полная потеря голоса. При двустороннем парезе пациентов больше беспокоит нарушение дыхания в виде инспираторной одышки и инспираторного стридора; голос же может быть звучным при срединном положении голосовых складок, охриплость характерна для параи интермедианного положения парализованных голосовых складок.

Прогнозируя риск повреждения ветвей гортанного нерва при хирургии ЩЖ или ОЩЖ, важно помнить о весьма вариабельной частоте возникновения осложнений в зависимости от причин и объема выполняемого оперативного вмешательства. Парез возвратного нерва и дисфония наиболее ожидаемы после оперативных вмешательств в объеме субтотальной или тотальной тиреоидэктомии, особенно дополненной лимфодиссекцией. В течение последних 10 лет наблюдается тенденция к снижению частоты хирургических осложнений при лечении заболеваний ЩЖ. В исследовании R. Bellantone и соавт., включавшем 526 пациентов после тотальной тиреоидэктомии, частота стойкого паралича возвратного нерва и стойкой гипокальциемии составила 0,4 и 3,4% соответственно [11].

Послеоперационный гипопаратиреоз — основное осложнение после операций по поводу заболеваний ЩЖ или ОЩЖ с развитием стойкого или рецидивирующего спазма голосовых связок из-за выраженного снижения уровня сывороточного кальция, вплоть до развития гипокальциемического криза. Это состояние может быть пропущено вследствие недостаточного контроля за уровнями кальция (общего и ионизированного) и паратиреоидного гормона (ПТГ) как в послеоперационном периоде, так и в более поздние сроки. Как показало исследование S. Viana Baptista и соавт. [12], субъективное и объективное исследования функций гортани в предоперационном периоде целесообразны для оценки динамики основного заболевания и послеоперационных осложнений, ранней реабилитации и дифференциального диагноза с заболеваниями, напрямую не связанными с эндокринопатией. Авторы призывают не концентрироваться только лишь на анатомии и сохранении функции возвратного нерва. По результатам исследования у большинства пациентов в той или иной степени были жалобы на изменение качества голоса или выявлены бессимптомные функциональные, реже органические изменения гортани в предоперационном периоде (утвердительные ответы на прямые вопросы о дисфонии — 16,7%; нарушения по опроснику Voice Handicap Index (VHI) легкой степени — 95,5%, средней степени — 4,9%; по шкале GRBAS (G = grade, R = roughness, B = breathiness, A = asthenia, and S = strain) более 1 — 35,8%; стробоскопия, отличающаяся от нормы, — 59,6%) при отсутствии нарушения подвижности гортани, что, вероятно, связано с медленным развитием патологии ЩЖ и адаптацией пациента к голосовым симптомам и/или наличием сопутствующей патологии гортани. При проведении предоперационной оценки голоса можно выявить ятрогенное повреждение или обострение имеющегося ранее заболевания в постоперационном периоде [12]. По данным D. Bartsch et al. на этапе предоперационной ларингоскопии из 12 578 пациентов с доброкачественным образованием ЩЖ у 116 выявлен односторонний парез гортани, у 4 — двусторонний [13].

Отечественными клиническими рекомендациями Российской ассоциации эндокринологов и Национальной медицинской ассоциации оториноларингологов, руководствами иностранных профессиональных сообществ (American Thyroid Association, American Academy of Otolaryngology-Head and Neck Surgery) рекомендуется осмотр голосовых складок у пациентов перед оперативным вмешательством на ЩЖ. Частота предоперационного пареза или паралича гортани у пациентов с доброкачественным заболеванием ЩЖ при предоперационной ларингоскопии колеблется от 0 до 3,5 и до 8 у пациентов с раком ЩЖ (10–15% случаев рака ЩЖ проявляются экстратиреоидным распространением) [14]. American Academy of Otolaryngology-Head and Neck Surgery в клинических рекомендациях по улучшению голоса после операций на ЩЖ настоятельно рекомендуется направлять пациентов к ларингологу, если симптомы сохраняются от 2 нед до 2  мес [15]. Прогноз и эффективность консервативного лечения напрямую зависят от сроков начала последнего, динамического контроля дыхательной и голосовой функции в послеоперационном периоде. Схема лечения при травматическом парезе гортани включает парентеральное введение глюкокортикостероидов, ангиопротекторов, витаминов группы В и антихолинэстеразных препаратов [14][15]. Степень стенозирования просвета гортани и, соответственно, тяжести состояния больного определяется при общем осмотре и проведении общеклинического обследования. При двустороннем парезе гортани нарушается дыхательная функция с развитием стеноза, неподвижные голосовые складки чаще находятся в срединном (дисфония отсутствует) или парамедианном положении, при котором несмыкание голосовых отростков черпаловидных хрящей 1–2 мм не вызывает значимых для пациента нарушений голоса. При одностороннем парезе дисфония проявляется при парамедианном, интермедианном или латеральном положении неподвижной складки. Кроме того, отмечен посттиреоидэктомический синдром, причины которого еще необходимо изучать, когда жалобы на изменение голоса и чувство кома в горле возникают в послеоперационном периоде без повреждения возвратного нерва и при нормальных показателях объективного исследования (ларингоскопия, акустический анализ, электромиография). Вероятно, при отсутствии каких-либо повреждений преходящие голосовые симптомы связаны с нормальным процессом заживления: функциональный компонент связан с местной болью и эмоциональной реакцией на операцию. Стойкие изменения голоса чаще наблюдались у пожилых женщин при тиреоидэктомии [10].

В исследовании L. Junuzović-Žunić и соавт. было выявлено достоверное различие до и после лечения гипотиреоза по акустическим параметрам и параметрам восприятия (шкала GRBAS), в отношении гипертиреоза — только максимальное время фонации имело значимую разницу и параметры восприятия [16].

При акромегалии — тяжелом системном, медленно прогрессирующем, нейроэндокринном заболевании — изменения голоса являются далеко не ведущими и проявляются не сразу. Высота формантных частот зависит от строения и размеров голосового тракта, языка и костей лицевого скелета. Хронический избыток соматотропного гормона у лиц с законченным физиологическим ростом опосредованно, через гиперпродукцию инсулиноподобного фактора роста-1, приводит к системным патологическим изменениям, среди которых основными являются диспропорциональный периостальный рост костей, хрящей, мягких тканей и внутренних органов, также нарушения морфофункционального состояния сердечно-сосудистой и легочной систем, работы других периферических эндокринных желез. Костные изменения черепно-лицевой области, увеличение объема верхнечелюстных пазух, пролиферация тканей верхних дыхательных путей, в том числе утолщение хрящей гортани и голосовых складок, соответствующим образом отражаются на голосовой функции в виде снижения высоты голоса, охриплости. При данной патологии также велика вероятность кальцификации и дислокации черпаловидных хрящей, ведущих к дисфонии [17]. Сочетание особенностей внешнего вида больного с рядом клинических проявлений дают основание заподозрить у пациента акромегалию. Но несмотря на то что акромегалия характеризуется выраженной симптоматикой, эти изменения нарастают постепенно, и для их проявления могут потребоваться годы. Поэтому при медленно прогрессирующем развитии акромегалии изменение характеристик голоса зачастую не отмечается ни самим пациентом, ни его родственниками. По данным исследования L.C. Wolters и соавт., лечение акромегалии (адъювантное консервативное + хирургическое или консервативное, при противопоказаниях к хирургическому) приводит к снижению баллов физиологического и физического компонентов опросника VHI, особенно в течение первого года лечения, причем физиологический компонент снижается больше у мужчин. Общий балл VHI также снизился, но статистически не значимо. Отмечалось уменьшение отека и гипертрофии слизистой оболочки при видеоларингостробоскопии, однако объективные параметры голоса остались неизменными: это объяснялось авторами возможной недостаточной чувствительностью аппаратуры при небольшом сдвиге параметров, компенсаторными изменениями, которые могли повлиять на восприятие голоса, и адаптацией пациентов к собственному голосу. Нужно учитывать, что средние значения, по данным опросника VHI, и до лечения находились в пределах нормальных значений, F0 — на нижней границе нормального диапазона [17].

При дефиците соматотропного гормона, в связи с малыми размерами голосового тракта, характерны более высокие значения формантных частот и длительное поддержание препубертатных акустических параметров голоса [1].

У девочек и женщин с синдромом Шерешевского– Тернера отмечается более высокий голос в связи с меньшими размерами гортани и частичной андрогенной недостаточностью, также характерным является носовой оттенок голоса, обусловленный строением речевого аппарата — высокое «готическое небо». Адекватная терапия препаратами гормона роста и половыми стероидами снижает частоту голоса и увеличивает его глубину [1].

С позиций синдрома гипогонадизма дисфонии делятся на характерные для первичного (преи постпубертатный гипогонадизм) и вторичного. Повышение уровня пролактина на фоне аденомы гипофиза, подавляющего секрецию лютеинизирующего гормона, — одна из причин вторичного гипогонадизма, клиническим проявлением которого у женщин является аменорея или дисменорея, поэтому возможны симптомы дисфонии, подобные менопаузальным физиологическим изменениям (описание которых подробнее представлено далее). Для мужчин характерно снижение силы и диапазона голоса, высота чаще неизменна. Также сила мужского голоса снижается при уменьшении уровня фолликулостимулирующего гормона и тестостерона [18]. Частным примером может быть и синдром гипогонадизма у мужчин с сахарным диабетом, особенно на фоне ожирения [19]. Однако, судя по изученным литературным источникам, жалобы на изменение голоса не являются значимыми в данных клинических ситуациях.

В рамках синдрома гиперандрогении описаны изменения голоса при избытке половых гормонов — синдроме поликистозных яичников (СПЯ), врожденной дисфункции коры надпочечников (ВДКН).

ВДКН характеризуется избыточным образованием андрогенов, что, вызывая вирилизацию голосового аппарата, может снизить F0 у женщин. Сходные изменения выявляются при повышении адренокортикотропного гормона на фоне аденомы гипофиза [1].

СПЯ, включающий гиперандрогению, наблюдается у 5–15% женщин репродуктивного возраста. Авторы одних исследований выявляют у данных пациенток больше голосовых симптомов по сравнению с контрольной группой, авторы других — не отмечают влияния СПЯ на голос [20][21].

Андрогены изменяют гистологическую структуру тканей голосовых складок, включая гипертрофию щиточерпаловидных мышц, паракератоз эпителия и железистую гиперплазию, что не может быть определено при ларингоскопии. Однако даже при отсутствии жалоб и объективных акустических различий при фиброскопии и стробоскопии было отмечено более частое выявление бессимптомной гипертонусной дисфонии, неполного закрытия голосовой щели и нарушения вибрации голосовых складок в группе пациентов с СПЯ по сравнению с контрольной группой. Данное явление может быть связано с повышенным уровнем андрогенов или тревожными и депрессивными расстройствами, риск которых ассоциирован с СПЯ [22].

При сахарном диабете изменения голоса могут наблюдаться как при остром гипергликемическом состоянии (особенно гиперосмолярном), так и как следствие хронической гипергликемии в виде диабетической нейропатии [23]. Гипергликемия, изменение баланса жидкости и электролитов при сахарном диабете могут косвенно влиять на голос. Типичной жалобой является ксеростомия, которая затрудняет фонацию в связи с нарушением увлажнения слизистой оболочки гортани. Диабетическая нейропатия нередко нарушает работу мышц гортани, участвующих в голосообразовании [1]. При остром гипергликемическом состоянии особенностью клинической картины является полиморфная неврологическая симптоматика (судороги, дизартрия, гиперили гипотонус мышц, парезы), не укладывающаяся в какой-либо четкий синдром, изменчивая и исчезающая при нормализации осмолярности. При достижении компенсации углеводного обмена, в случае отсутствия полинейропатии с нарушением работы мышц гортани, изменения голоса обратимы [23]. В исследовании A. Hamdan и соавт. отмечена взаимосвязь между неадекватным гликемическим контролем и охриплостью [24].

Пациенты с нейросенсорной тугоухостью на фоне прогрессирующего сахарного диабета склонны говорить с повышенной интенсивностью, что может отрицательно сказаться на состоянии голосовых складок. Кроме того, внепищеводные проявления гастроэзофагеальной рефлюксной болезни часто встречаются у пациентов с сахарным диабетом и ассоциируются с плохим гликемическим контролем, вегетативной нейропатией, ожирением и использованием лекарств, влияющих на тонус нижнего сфинктера пищевода [1].

Эндокринные расстройства, описанные выше, и ряд других нередко сопровождаются изменением голоса, однако при грамотном консервативном и хирургическом лечении основного заболевания возможно полное его восстановление. При этом физиологические возрастные изменения оказывают значительное влияние на голос даже относительно здоровых людей, что особенно актуально для певцов, актеров, учителей и других представителей голосоречевых профессий.

Половые гормоны, наряду с гормонами ЩЖ, наибольшим образом воздействуют на голосовой аппарат человека. Вопрос влияния половых гомонов на голос, являющийся важным вторичным половым признаком, требует особого внимания. Повышение уровня андрогенов у юношей во время полового созревания приводит к увеличению объема мышц, связок и размеров хрящей гортани, что ведет к снижению основного тона голоса на одну-полторы октавы. Дети не успевают перестроить корковые импульсы на функционирование в новых условиях, что приводит к дискоординации функционирования голосового аппарата, обычно физиологическая мутация завершается адаптацией [25]. Однако возможна патологическая мутация в виде задержки мутации, неполной мутации, преждевременной мутации, мутационного фальцета. Первичный гипогонадизм может стать причиной развития щитовидного хряща и прочих структур гортани у мужчин по женскому типу, и, соответственно, затянувшейся мутационной дисфонии, которая характеризуется высоким основным тоном, слабостью голоса, охриплостью, диплофонией. Коррекция проводится при помощи фонопедии, при ее неэффективности — инъекций ботулотоксина в надподъязычные мышцы либо хирургического вмешательства параллельно с лечением основного состояния [26].

Женский голос не претерпевает столь резких изменений на фоне повышения уровня эстрогенов и гестагенов в пубертатный период, меняется на 3–4 полутона. Однако значительное гормональное влияние на женский голос можно оценить, основываясь на изменениях, связанных с менструальным циклом. Во время беременности женщина сталкивается со множеством физических и эмоциональных изменений. На фоне повышения уровня эстрогена, прогестерона и пролактина отмечается отек голосовых складок, наблюдаются расширение сосудов голосовых складок, ухудшение ларингофарингеального рефлюкса, изменение осанки, снижение емкости легких, изменение резонанса. В исследовании H. Ghaemi и соавт. выявлены значимые отличия в виде отека голосовых складок, уменьшения максимального времени фонации (МВФ) и изменений по данным VHI лишь в III триместре беременности. Снижение МВФ может быть связано с изменением объема брюшной полости и емкости легких [27]. Ряд других исследований [28][29] также подтверждает изменения структуры голосовых складок в виде увеличения толщины эпителия, сухости, вязкости секрета и снижения МВФ в III триместре беременности. Практически 1/3 женщин репродуктивного возраста, не использующих голос профессионально, отмечают изменение голоса в течение жизни, а также значительные изменения в постменопаузе [22]. В понятие предменструального синдрома также входит дисфония [1]. Физиологические изменения женского голоса в течение жизни резюмированы в таблице 1. Даже если при слуховой оценке голос признается нормальным, следует отнестись к жалобам внимательно, так как в дальнейшем у этих женщин может развиться гипотонус голосовых складок или вазомоторный монохордит [30]. Заместительная гормональная терапия может способствовать менее заметным проявлениям пресбифонии (понижения частотных характеристик), наблюдаемым в постменопаузе.

**Table:** Таблица 1. Физиологические изменения голосового аппарата и характеристик голоса у женщин в течение жизни по K.V.S. Hari Kumar и соавт. (2016) [1]Table 1. Physiological changes in the vocal apparatus and characteristics of the voice in women throughout life according to K.V.S. Hari Kumar et al. (2016) [1]

Фаза жизненного цикла	Гормональные изменения	Структурные изменения	Изменение характеристик голоса
Половое созревание	Увеличение уровней эстрогенов и гестагенов	Рост голосовых складок и прочих структур голосового аппарата	Отсутствуют значимые изменения
Менструальный цикл
Фолликулярная фаза	Увеличение уровня эстрогенов	Отечность структур гортани	Снижение выносливости голоса
Овуляторная фаза	Увеличение уровня эстрогенов	Увеличение толщины голосовых складок	Затруднение при пении высоких нот
Лютеиновая фаза	Увеличение уровня прогестерона	Повышение вязкости секрета желез гортани, нарушение увлажнения ее слизистой оболочки. Снижение тонуса мышц гортани	Сужение диапазона голоса
Менопауза	Снижение уровней эстрогенов и прогестерона.Увеличение уровня андрогенов	Атрофия мышц гортани.Оссификация хрящей гортани.Увеличение толщины и снижение эластичности голосовых связок	Снижение силы и выносливости голоса. Сужение диапазона

Нередко к естественному физиологическому старению люди относятся как к неизбежному процессу, который, хотя и снижает качество жизни, не требует дополнительной профилактики и лечения. При этом в популяции людей старше 65 лет в 30% случаев выявляется дисфония [31]. Однако важно помнить, что с уверенностью поставить диагноз «пресбифония» можно менее чем в 10% случаев, так как с возрастом многие люди приобретают различные заболевания гортани, проявляющиеся расстройством голоса: ларингиты, нодозные образования голосовых складок, рак гортани, парезы и параличи голосовых складок и прочие [32].

Организм человека подвергается гормональным и общим инволюционным изменениям, среди которых — атрофия мышечного и связочного аппарата гортани, кальцификация хрящей, снижающая их подвижность; уменьшение эластичности скелета грудной клетки и жизненной емкости легких, что укорачивает фонационный выдох и снижает силу голоса; атрофия железистых структур и лимфоидно-глоточного кольца Пирогова–Вальдейера, приводящая к нарушению увлажнения слизистой оболочки гортани, снижению местного иммунитета; увеличение площади распространения многослойного плоского эпителия, разрастание соединительной ткани и жировой клетчатки, увеличение плотности голосовых складок. При ларингоскопии голосовые складки атрофичны, немного провисают, свободный край неровный. Дегенеративные изменения приводят к снижению амплитуды колебательных движений голосовых складок и неполному их смыканию при фонации. Это способствует изменению высоты голоса и появлению добавочных призвуков в голосе — охриплости, придыхания, дрожания. Стоит также учитывать наличие сопутствующих соматических заболеваний, как возможную причину пресбифонии [31].

У лиц с пресбифонией выявляются изменения практически всех акустических параметров голоса. Женский голос становится ниже, уменьшаются его сила и выносливость, сужается динамический и частотный диапазон за счет его верхней части. У мужчин также происходит сужение частотного диапазона, голос становится выше, но динамический диапазон практически не меняется [32]. Известно, что возрастные изменения голоса у представителей голосоречевых профессий происходят медленнее и в более позднее время [31].

В целях профилактики возрастных изменений голоса рекомендованы модификация образа жизни, физиотерапия, лечебная физкультура и фармакотерапия, включающая ноотропные препараты, стимуляторы процессов тканевого обмена и витаминно-минеральные комплексы, включающие витамины А, группы В и цинк. Большую роль в профилактике и коррекции пресбифонии играет речевая и вокальная фонопедия [31].

## ЗАКЛЮЧЕНИЕ

Этиопатогенетический подход к лечению основного заболевания делает возможным восстановление голосовой функции при описанных в статье эндокринных нарушениях. Отечественными и зарубежными руководствами профессиональных сообществ рекомендуется проведение видеоларингоскопии у пациентов как с нормальным голосом, так и с голосовыми нарушениями перед оперативным вмешательством на ЩЖ и интраоперационного нейромониторинга возвратных гортанных нервов [33][34]. Рекомендуется направлять пациента к оториноларингологу и фониатру для проведения ларингостробоскопии и оценки качества жизни и голоса пациентов с помощью валидизированного опросника VHI и шкалы GRBAS, а также последующего длительного наблюдения и лечения [33][35]. Данные опросники и шкалы используются также при акромегалии, гипогонадизме и других патологиях.

До операции на ЩЖ пациента необходимо предупредить о возможных послеоперационных нарушениях голоса и дыхания; при возникновении последних обязательной является консультация оториноларинголога с осмотром гортани и оценкой подвижности голосовых складок [33].

После хирургических вмешательств на ЩЖ при одностороннем повреждении возвратного нерва проводят следующее лечение: глюкокортикостероиды парентерально, ангиопротекторы, витамины группы В и антихолинэстеразные препараты; электростимуляцию синусоидально-модулированными токами и электрофорезом с прозерином на область гортани, рефлексотерапию и нейромышечную электрофонопедическую стимуляцию в комбинации с дыхательной гимнастикой. Последние делают возможным улучшение голосовой функции даже при стойком параличе — за счет компенсаторного захождения функционирующей голосовой складки за среднюю линию и смыкания ее с парализованной. В случае положительной динамики голосовой функции после консервативного лечения больным рекомендуется проводить курс фонопедических упражнений [14][33][36].

При односторонних параличах гортани, в случае отсутствия эффективности от проведения консервативного лечения, используют следующие виды хирургических вмешательств: имплантация различных веществ в голосовую складку — эндоларингеальная инъекционная медиализация голосовой складки; наружная тиреопластика — приведение парализованной голосовой складки с помощью различных имплантов (аутохрящ, тефлон) к средней линии через окно в пластине щитовидного хряща [33][37].

При двустороннем посттравматическом парезе гортани на раннем этапе лечения консервативное лечение пациентов должно проводиться в условиях лор-стационара; нельзя проводить стимулирующие физиопроцедуры и фонопедическую коррекцию, так как это может привести к сужению просвета голосовой щели и декомпенсации стеноза за счет активации приводящих мышц гортани [33][36]. При декомпенсации стеноза гортани показана трахеотомия для восстановления дыхательной функции с последующим консервативным лечением.

При отсутствии восстановления подвижности голосовых складок в течение 12  мес показано реконструктивно-пластическое вмешательство на структурах гортани по расширению просвета голосовой щели [33].

При физиологических изменениях голоса — корректное и внимательное общение с пациентами позволит клиницистам определить наиболее эффективные и безопасные методы реабилитации голосовой функции с учетом сопутствующей патологии, индивидуальных особенностей организма и личности.

## ДОПОЛНИТЕЛЬНАЯ ИНФОРМАЦИЯ

Источники финансирования. Работа выполнена по инициативе авторов без привлечения финансирования.

Конфликт интересов. Авторы декларируют отсутствие явных и потенциальных конфликтов интересов, связанных с содержанием настоящей статьи.

Участие авторов. Все авторы одобрили финальную версию статьи перед публикацией, выразили согласие нести ответственность за все аспекты работы, подразумевающую надлежащее изучение и решение вопросов, связанных с точностью или добросовестностью любой части работы.
